# Increased interleukin-9 and Th9 cells in patients with refractory Graves’ disease and interleukin-9 polymorphisms are associated with autoimmune thyroid diseases

**DOI:** 10.3389/fimmu.2024.1341749

**Published:** 2024-03-28

**Authors:** Qiuming Yao, Zhenyu Song, Bin Wang, Peng Du, Qiu Qin, Jing Zhao, Jin-an Zhang

**Affiliations:** ^1^ Department of Endocrinology, Shanghai University of Medicine & Health Sciences Affiliated Zhoupu Hospital, Shanghai, China; ^2^ Department of Tumor Interventional Oncology, School of Medicine, Renji Hospital, Shanghai Jiaotong University, Shanghai, China

**Keywords:** Hashimoto’s thyroiditis, IL-9, polymorphisms, refractory Graves’ disease, Th9 cells

## Abstract

**Introduction:**

Autoimmune thyroid diseases (AITDs) are prevalent disorders, primarily encompassing Graves’ disease (GD) and Hashimoto’s thyroiditis (HT). Despite their common occurrence, the etiology of AITDs remains elusive. Th9 cells, a new subset of CD4+T cells with immunomodulatory properties, have been linked to the development of various autoimmune diseases. However, research on the role of Th9 cells in AITDs is limited.

**Methods:**

We investigated the expression of Th9 cells,their functional cytokine IL-9, and transcription factor IRF4 in peripheral blood mononuclear cells (PBMCs) and plasma of AITD patients and healthy controls. Additionally, we explored the genetic association between four loci polymorphisms (rs31564, rs2069879, rs1859430, and rs2069868) of the IL-9 gene and AITDs.

**Results:**

We reported, for the first time, that refractory GD patients exhibited elevated mRNA levels of IL-9 and IRF4 in PBMCs, increased IL-9 protein levels in plasma, and a higher proportion of Th9 cells in peripheral blood when compared to normal controls. Furthermore, human recombinant IL-9 protein was found to enhance IFN-g secretion in PBMCs from both GD patients and normal controls. At the genetic association level, after adjusting for age and sex, the rs2069879 polymorphism exhibited a significant association with AITDs under an additive model (P<0.001, OR= 0.05, 95% CI=0.03-0.08).

**Discussion:**

Our results reveal that Th9 cells may exert a pivotal role in the pathogenesis and progression of refractory GD and HT, and IL-9 holds promise as a novel therapeutic target for the management of AITDs.

## Introduction

Autoimmune thyroid diseases (AITDs), with a 5% prevalence, involve a disruption of the immune system resulting in an autoimmune attack directed at the thyroid gland (1–3). AITDs mainly manifest as two subtypes, namely, Graves’ disease (GD) and Hashimoto’s thyroiditis (HT). The infiltration of lymphocytes into thyroid tissue is the shared pathological feature of the two diseases (1). Patients with GD typically present with hyperthyroidism, while those with HT commonly exhibit hypothyroidism.

It is now widely accepted that genetic, autoimmune and environmental factors are synergistically involved in the development of AITDs. Different subtypes of CD4^+^T cells can secrete specific cytokines and play a key role in regulating immune and autoimmune responses (4). After antigen stimulation, naive CD4^+^T cells can eventually differentiate into different cell subsets according to different stimulation conditions. The role and mechanism of CD4^+^T cell subsets in AITDs has received great attention in recent years. Various studies have found that they are related to the occurrence of many autoimmune diseases including AITDs. For example, Th17/Treg cells are in a state of balance, and when this balance is broken, it will promote the occurrence of autoimmune or inflammatory diseases (5). In AITDs patients, there is an increase in the Th17/Treg ratio than healthy controls (6).

At the end of 2008, two research groups, led by Darhalhon (7) and Vekdhoen (8), almost simultaneously found in a mouse model that co-culture of TGF-β and IL-4 could induce the generation of a new CD4^+^T cell subset, named Th9 cells, which could play an immunomodulation role by secreting IL-9. IL-9 is a versatile cytokine that acts on a variety of cell types and different tissues (9), whereas IRF4 serves as a crucial transcription factor necessary for Th9 cell differentiation. In recent years, it has been found that Th9 cells are related to the occurrence of many autoimmune diseases. The expressions of Th9 cells and IL-9 are increased in the mucosal tissues of ulcerative colitis (UC) patients and peripheral blood of systemic lupus erythematosus (SLE) patients and are related to the activity and severity of SLE (10, 11). Th9 cells also exert a proinflammatory effect in the occurrence of rheumatoid arthritis (RA), asthma, psoriatic arthritis and dermatitis (12–15). However, IL-9 can alleviate autoimmune neuroinflammation by regulating the function of dendritic cells (16). Also, studies have found that IL-9 can interfere with the expression of IL-17 in Th17 cells in multiple sclerosis, and is negatively correlated with inflammatory activity index and disease severity (17). Above findings indicate that Th9 cells play different roles in different diseases. Although recently published studies have demonstrated an increase in Th9 cells and IL-9 in newly diagnosed GD patients and iodine-induced autoimmune thyroiditis model (18, 19), but the role of Th9 cells in refractory GD and HT patients is still unclear. Therefore, in this study, we firstly explored the expression and the role of Th9 cells in them. Our research will provide one of AITDs pathogenic regulatory mechanisms and be helpful to seek the novel potential immunotherapeutic target for AITDs, the pivotal diseases in the polyautoimmune disorders, namely the type 3 polyglandular autoimmune disorder (20).

In this study, we firstly investigated the expression of Th9 cells, IL-9 and IRF4 in refractory GD patients and HT patients. We also explored the relationship between Th9 cells and Th1, Th2, th17 and Th22 cells, and analyzed the association of IL-9 locus (rs31564, rs2069879, rs1859430 and rs2069868) polymorphism with AITDs.

## Materials and methods

### Subjects

The inclusion criteria of GD were as follows: clinical manifestations of thyrotoxicosis, elevated levels of free thriiodothyronine (FT3) and free thyroxine (FT4), decreased thyrotropic-stimulating hormone (TSH), positive thyrotropin receptor antibody (TRAb), and absence of other autoimmune diseases or acute/chronic diseases. Refractory or intractable GD patients were defined as those given anti-thyroid drug for a period of at least four years, yet still showed a positive result for TRAb (21). HT inclusion criteria: positive antibodies to thyroglobulin (TgAb) or antibodies to thyroperoxidase (TPOAb), diffuse hypoechoic changes in thyroid parenchyma by B-ultrasound and absence of other autoimmune diseases or acute/chronic diseases. Normal controls (NC) inclusion criteria: no thyroid-related diseases, other autoimmune diseases or family history of autoimmune diseases, or infectious diseases or genetic diseases. To exclude other autoimmune disorders, we conducted a thorough interview, medical history review and physical examination for each participant in our study. Patients with a known history or clinical evidence suggestive of these disorders were excluded from our analysis to minimize confounding factors of our results. In addition, we use appropriate laboratory assessments such as antinuclear antibodies, where appropriate, to screen for the presence of other autoimmune diseases, as ANA is present in many connective tissue diseases. But we should note that the measurement of circulating autoantibodies is not sufficient to exclude the presence of an auto aggressive disorder, mainly in those that are subclinical as gastric disorders (22).

A total of 1156 AITDs patients, consisting of 719 GD patients and 437 HT patients, and 791 normal controls were recruited. PBMCs from 38 GD patients, 24 HT patients and 24 NC were collected for PCR assay. Plasma was collected from 24 GD, 23 HT and 21 NC for the protein chip of cytokine detection. PBMCs from 28 GD, 23 HT and 16 NC were used for flow cytometry analysis. PBMCs from 6 GD, 5 HT and 6 NC were collected for cell culture. The remaining 985 AITDs patients (623 GD and 362 HT) and 724 NC were included for the single nucleotide polymorphism (SNP) detection. The number of samples in each part of the experiment was summarized in **Supplementary Table 1**. All subjects were recruited from the Department of Endocrinology of Zhoupu Hospital, affiliated with Shanghai University of Medicine & Health Sciences.

### Quantitative real-time polymerase chain reaction

Total mRNA was extracted from PBMCs using Trizol reagent (Takara, Japan), and cDNA was synthesized with a reverse transcription kit (Takara) following the manufacturer’s protocol. The mRNA expression levels of IFN-γ, IL-4, IL-10, IL-9, IRF-4, IL-17, IL-21, and IL-22 were assessed, using the SYBR Premix Ex Taq TMII kit (TaKaRa) on a qRT-PCR analyzer, with β-actin as the internal control gene. **Supplementary Table 2** provides primer sequences of each gene.

### Flow cytometric analysis

A 2μL combination (BD Bioscience, USA) of phorbol myristate acetate, ionomycin and monensin was used to stimulate PBMCs for 4 hours. Then, to stain PBMCs with anti-human CD4-FITC (BD Pharmingen, USA), they were placed in a dark environment at 4^°^C for 30 minutes, and the Cytofix/Cytoperm kit (BD Biosciences, USA) was used to conduct fixation and permeabilization. After that, these cells were incubated under a light-free environment at 4^°^C with anti-human IL-9-APC (BD Pharmingen, USA) for 30 minutes. The Th9 cells (CD4^+^IL-9^+^T cells) were immediately analyzed using the FACScalibur Flow cytometer (Beckman coulter).

### Cell culture

Individuals with GD, HT, and NC donated fresh peripheral blood samples. Then PBMCs were isolated, counted (1∼2× 10^6^/ml), and plated into 6-well plates. Two separate collections of samples were generated. One group was incubated with a combination of recombinant IL-2 protein(50 ng/ml) from PeproTech (USA) and recombinant IL-9 (100 ng/ml) from R&D Systems (USA), while the other group was stimulated only with IL-2. Following a 48-hour incubation, cell supernatants were collected. The IFN-γ, IL-5, IL-13, IL-17 (IL-17A), IL-22, and IL-10 protein levels in the cell supernatant were measured using the Human High Sensitivity T Cell Magnetic Bead Panel (Merck Millipore, Germany), following the manufacturer’s protocol.

### SNP genotyping

Four loci (rs31564, rs2069879, rs1859430, rs2069868) of IL-9 gene were selected by searching literature and NCBI database. SNP selection criteria were as follows: 1) SNPs that had not been studied in Chinese Han AITDs patients (Rs1859430 and rs2069868 had only been studied in Chinese Han GD but not in HT); 2) minor allele frequency >0.01; 3) compliance with Hardy-Weinberg equilibrium (HWE) (P>0.05). High throughput-SNP sequencing, a method developed by Shanghai Biowing Applied Biotechnology Inc., was employed to detect the genotype of DNA samples. To check the inconsistent genotyping results, Sanger sequencing analysis was done on the samples, and only those that achieved the 95% quality control standard were taken into account for further study.

### Statistical methods

Data from gene expression, flow cytometry and the *in vitro* PBMCs culture fraction were analyzed using Graphpad Prims5.0. Continuous variables are presented as means ± SE. Depending on the nature of the data, the Mann-Whitney U test or t-test was used for analyzing group differences. Spearman method was used to analyze the correlation between the two indicators. Changes in the concentration of each cytokine in the cell culture supernatant were analyzed by paired t test or Wilcoxon method. The chi-square test was utilized for calculating genotype and allele frequency differences using SPSS17.0. Using Stata 12.0, we used multivariate logistic regression to assess the correlation between IL-9 polymorphisms and AITDs under alleles (c vs C), dominant (cc+Cc vs CC), and additional models (Cc vs CC). The cutoff point for statistical significance was fixed at P< 0.05.

## Results

### Increased mRNA expressions of IL-9 and IRF4 in PBMCs of AITDs patients

**Figure 1:**
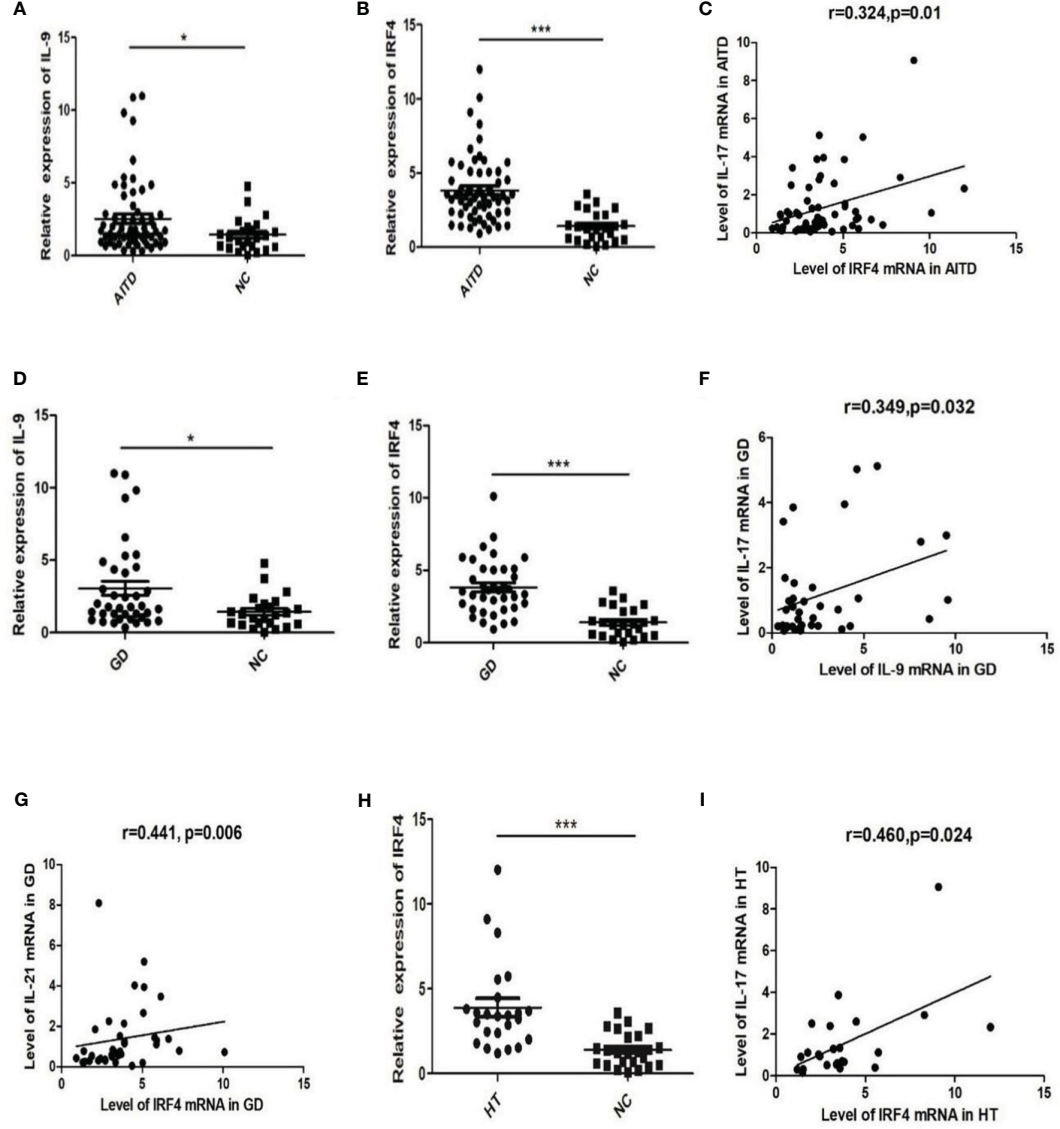
mRNA expression of IL-9 and IRF4 in AITD patients and correlation analysis of IL-9 and IRF4 mRNA and level of Th17 related cytokines mRNA expression.**(A, B)** Relative expression of IL-9 **(A)** and IRF4 **(B)** in AITD group; **(C)** Correlation analysis of IRF4 mRNA and IL-17 mRNA in AITD group; **(D, E)** Relative expression of IL-9 **(D)** and IRF4 **(E)** in GD group; **(F)** Correlation analysis of IL-9 mRNA and IL-17 mRNA in GD group; **(G)** Correlation analysis of IRF4 mRNA and IL-21mRNA in GD group; **(H)** Relative expression of IRF4 in HT group; **(I)** Correlation analysis of IRF4 mRNA and IL-17 mRNA in HT group. *P<0.05; ***P<0.001.

As shown in **Figure 1**, mRNA expressions of IL-9 and IRF4 in the AITDs group significantly exceeded those in the NC group (P=0.03 and P<0.0001, respectively, **Figures 1A, B**). Correlation analysis did not reveal any significant correlations between IL-9 mRNA expression and IFN-γ, IL-4, IL-17, IL-10, IL-21, or IL-22 mRNA expressions in AITDs group PBMCs (all P > 0.05) (data not shown). IRF4 mRNA expression was significantly linked with IL-17 mRNA expression (r=0.324, P=0.01, **Figure 1C**), but not with IFN-, IL-4, IL-10, IL-21, or IL-22 mRNA expression (all P > 0.05, data not shown).

Subgroup analysis revealed higher relative mRNA expression levels of IL-9 and IRF4 in GD patients’ PBMCs than in the NC group (P=0.013 and P<0.001, **Figures 1D, E**). However, these expression levels had no correlation with TRAb (all P>0.05, data not shown). In the GD group, relative mRNA expression of IL-9 and IL-17 showed a strong correlation (r=0.349, P=0.032, **Figure 1F**). The IRF4 mRNA expression was similarly favorably linked with the level of IL-21 mRNA (r=0.441, P=0.006, **Figure 1G**).

The relative mRNA expression of IRF4 was dramatically elevated in the HT group (P 0.001, **Figure 1H**). A positive association was found between the relative expression levels of IRF4 mRNA and IL-17 mRNA (r=0.460, P=0.024, **Figure 1I**). The relative expression level of IL-9 mRNA in PBMCs did not differ significantly between the HT and NC groups (P > 0.05, data not shown).

### Elevated IL-9 protein levels in plasma of AITDs patients

**Figure 2:**
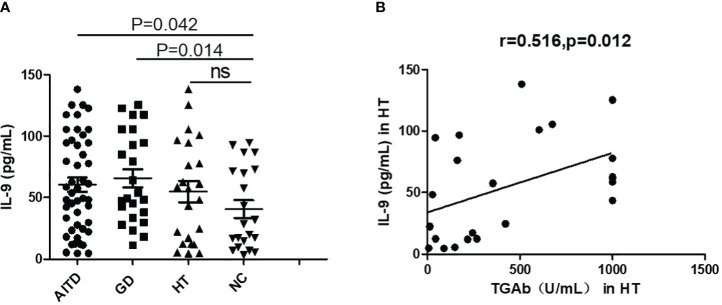
Plasma IL-9 protein levels in AITD patients. **(A)** Plasma IL-9 protein levels in AITD, GD, HT and NC groups. **(B)** Correlation analysis of plasma IL-9 protein levels and TGAb of HT patients. TGAb, antibodies to thyroglobulin.

As depicted in **Figure 2**, IL-9 levels in the blood were substantially higher in AITDs group than in the NC group (P=0.042, **Figure 2A**). Subgroup analysis revealed that the GD group had greater plasma IL-9 levels than the NC group (P=0.014, **Figure 2A**), whereas the HT group simply had a growing trend (P > 0.05). IL-9 level correlated positively with TgAb (r=0.516, P=0.012, **Figure 2B**) but not with TPOAb (P > 0.05, data not shown) in HT patients. In the GD group, IL-9 had no relationship with FT3, FT4, or TRAb (all P > 0.05, data not shown).

### Higher percentage of Th9 Cells in PBMCs of AITDs patients

**Figure 3:**
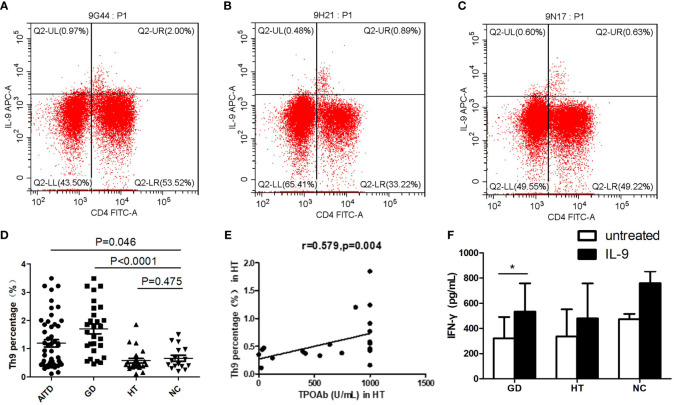
Expression of Th9 cells in PBMCs of AITD group and Expression of cytokines in supernatants of PBMCs from AITD patients after IL-9 stimulation. **(A–C)** Flow cytometry was used to detect the expression of Th9 cells in the PBMCs of GD, HT and NC group. **(D)** the expression difference of Th9 cell percentage among AITD group, GD group, HT group and NC group; **(E)** correlation analysis of the percentage of Th9 cells in PBMCs and TPOAb in HT group; **(F)** IFN-γ expression level in the supernatant of PBMCs in GD, HT and NC group. PBMCs in each group were stimulated with IL-2 (untreated) or human recombinant IL-9 protein combined with IL-2 for 48 hours, respectively. TPOAb, antibodies to thyroperoxidase (TPOAb). * P<0.05.

Flow cytometry was used to determine the Th9 cells (CD4+IL-9+T cells) proportion in the PBMCs of AITDs and NC group (**Figures 3A–C**), and the link between the proportion of Th9 cells and clinical indicators in the GD and HT groups was also investigated. Th9 proportion in the AITDs group’s PBMCs was considerably greater than in the NC group (P=0.046, **Figure 3D**). Subgroup analysis revealed that the GD group PBMCs had a considerably higher percentage of Th9 cells than the NC group (P<0.001, **Figure 3D**). In comparison to the NC group, the Th9 proportion in the HT group’s PBMCs increased, albeit not statistically significantly (P > 0.05). But in the HT group, the Th9 percentage and TPOAb concentration showed a significant positive correlation (r=0.579, P=0.004, **Figure 3E**). However, there was no link between Th9 percentage and FT3, FT4, or TRAb concentration (all P > 0.05, data not shown).

### Increased IFN-γ in PBMCs supernatant of GD group after IL-9 stimulation

The results showed that after recombinant IL-9 stimulation, the concentration of IFN-γ in the supernatant of PBMCs from both GD and NC groups considerably increased (P=0.031; P=0.031, respectively) (**Figure 3F**). Meanwhile, the HT group’s PBMC supernatant encompassed more IFN-γ than before stimulation, although the difference was not significant (P > 0.05). After recombinant IL-9 stimulation, the IL-5, IL-10, IL-13, IL-17 and IL-22 concentrations in the supernatant of PBMCs in the GD, HT and NC groups, were not significantly changed (all P > 0.05, data not shown).

### Association between IL-9 polymorphism and AITDs

The allele or genotype distributions of the four SNPs did not differ between AITDs, GD, and HT patients and NC group (all P > 0.05) (**Table 1**; **Supplementary Tables 3**-**5**). The IL-9 rs2069879 polymorphism was significantly associated with AITDs (P<0.001, OR=0.05, 95% CI=0.03-0.08) under the additive model. After adjustment for gender and age, the association still existed (P<0.001, OR=0.05, 95% CI=0.03-0.08) (**Table 2**). Subgroup analysis showed that IL-9 rs2069879 polymorphism was statistically linked to GD under the additive model (before adjustment: P<0.001, OR=0.048, 95% CI=0.03-0.08; adjusted for sex and age: P<0.001, OR=0.047, 95% CI=0.03-0.08) (**Table 3**). Also under the additive model, IL-9 rs2069879 polymorphism was significantly associated with HT (before adjustment: P<0.001, OR=0.05, 95%CI=0.03-0.09; adjusted for sex and age: P<0.001, OR=0.05, 95%CI=0.03-0.10) (**Table 4**).

**Table 1:** Genotype distributions of IL-9 loci in AITD patients and controls.

SNP	Genotype	NC (%)	AITD (%)	P	GD (%)	P	HT (%)	P
rs31564	TT	135 (18.6)	197 (20.0)	0.783	120 (19.3)	0.801	77 (21.3)	0.369
	TG	356 (49.2)	477 (48.4)		295 (47.4)		182 (50.3)	
	GG	233 (32.2)	311 (31.6)		208 (33.3)		103 (28.4)	
rs2069879	GG	1 (0.1)	0 (0)	0.352	0 (0)	0.427	0 (0)	0.716
	CG	57 (7.9)	67 (6.8)		41 (6.6)		26 (7.2)	
	CC	666 (92.0)	918 (93.2)		582 (93.4)		336 (92.8)	
rs1859430	AA	1 (0.1)	0 (0)	0.275	0	0.334	0 (0)	0.660
	AG	57 (7.9)	64 (6.5)		39 (6.3)		25 (6.9)	
	GG	666 (92.0)	921 (93.5)		584 (93.7)		337 (93.1)	
rs2069868	AA	18 (2.5)	23 (2.3)	0.974	16 (2.6)	0.994	7 (1.9)	0.811
	AG	173 (23.9)	238 (24.2)		148 (23.8)		90 (24.9)	
	GG	533 (73.6)	724 (73.5)		459 (73.6)		265 (73.2)	

**Table 2:** Association of three polymorphisms models in IL-9 with AITD before and after adjusting for age and sex.

Locus	Model	Adjust before		Adjust after	
		*P*	OR (95% CI)	*P*	OR (95% CI)
rs31564	Allele mode	0.570	1.04 (0.91-1.19)	0.731	1.02 (0.89-1.18)
	Dominant model	0.791	1.03 (0.84-1.26)	0.941	0.99 (0.80-1.22)
	Additive model	0.973	1.00 (0.81-1.25)	0.750	0.96 (0.77-1.21)
rs2069879	Allele mode	0.297	0.83 (0.56-1.18)	0.177	.0.78 (0.54-1.12)
	Dominant model	0.343	0.84 (0.58-1.21)	0.215	0.79 (0.54-1.45)
	Additive model	**<0.001**	**0.05 (0.03-0.08)**	**<0.001**	**0.05 (0.03-0.08)**
rs1859430	Allele mode	0.197	0.79 (0.55-1.13)	0.110	0.74 (0.51-1.07)
	Dominant model	0.231	0.80 (0.55-1.15)	0.136	0.75 (0.52-1.09)
	Additive model	0.271	0.81 (0.56-1.17)	0.168	0.77 (0.53-1.12)
rs2069868	Allele mode	0.989	0.99 (0.83-1.21)	0.740	1.03 (0.85-1.26)
	Dominant model	0.957	1.01 (0.81-1.25)	0.742	1.04 (0.83-1.30)
	Additive model	0.912	1.01 (0.81-1.27)	0.760	1.04 (0.82-1.31)

**Table 3:** Association of three polymorphisms models in IL-9 with GD before and after adjusting for age and sex.

Locus	Model	Adjust before		Adjust after	
		*P*	OR (95% CI)	*P*	OR (95% CI)
rs31564	Allele mode	0.879	0.99 (0.85- 1.15)	0.794	0.98 (0.84-1.14)
	Dominant model	0.639	0.95 (0.75-1.19)	0.539	0.93 (0.74-1.17)
	Additive model	0.547	0.93 (0.73-1.18)	0.459	0.91 (0.71-1.16)
rs2069879	Allele mode	0.280	0.80 (0.53- 1.20)	0.194	0.76 (0.50-1.15)
	Dominant model	0.317	0.81 (0.53-1.23)	0.227	0.77 (0.51-1.17)
	Additive model	**<0.001**	**0.048 (0.03-0.08)**	**<0.001**	**0.047 (0.03-0.08)**
rs1859430	Allele mode	0.189	0.76 (0.50-1.15)	0.128	0.72 (0.48-1.10)
	Dominant model	0.216	0.77 (0.50-1.17)	0.151	0.73 (0.48-1.12)
	Additive model	0.249	0.78 (0.51-1.19)	0.180	0.75 (0.49-1.14)
rs2069868	Allele mode	0.993	1.0 (0.81-1.24)	0.842	1.02 (0.83-1.27)
	Dominant model	0.981	0.99 (0.781/27)	0.892	1.02 (0.80-1.30)
	Additive model	0.959	0.99 (0.77-1.28)	0.944	1.01 (0.78-1.30)

**Table 4:** Association of three polymorphisms models in IL-9 with HT before and after adjusting for age and sex.

Locus	Model	Adjust before		Adjust after	
		*P*	OR (95% CI)	*P*	OR (95% CI)
rs31564	Allele mode	0.159	1.14(0.95-1.36)	0.170	1.14(0.94-1.38)
	Dominant model	0.210	1.19 (0.91-1.57)	0.341	1.15(0.86-1.54)
	Additive model	0.330	1.16(0.86-1.55)	0.579	1.09(0.80-1.49)
rs2069879	Allele mode	0.581	0.88(0.54-1.41)	0.274	0.76(0.47-1.24)
	Dominant model	0.630	0.89(0.55-1.44)	0.317	0.78(0.47-1.28)
	Additive model	**<0.001**	**0.05(0.03-0.09)**	**<0.001**	**0.05(0.03-0.10)**
rs1859430	Allele mode	0.476	0.84(0.52-1.36)	0.208	0.73(0.44-1.19)
	Dominant model	0.519	0.85(0.52-1.39)	0.243	0.74(0.45-1.23)
	Additive model	0.566	0.87(0.53-1.41)	0.286	0.76(0.46-1.26)
rs2069868	Allele mode	0.966	0.99(0.77-1.28)	0.639	1.07(0.82-1.39)
	Dominant model	0.884	1.02(0.77-1.36)	0.576	1.09(0.81-1.47)
	Additive model	0.763	1.05(0.78-1.40)	0.547	1.10(0.81-1.50)

## Discussion

The differentiation of Th9 cells can be synergistically induced by the combined action of TGF-β and IL-4, and this process necessitates the involvement of the transcription factor IRF4 (23). IRF4 knockout mice are unable to differentiate their T cells into Th9 cells as expected (24). The application of small interfering RNA to diminish IRF4 expression caused a significant reduction in the production of IL-9 from Th9 cells (25). Therefore, in addition to the major cytokine IL-9, we also examined the expression of IRF4 in all subjects. Our results revealed higher IL-9 and IRF4 mRNA levels in PBMCs, increased plasma IL-9 protein expression, and an elevated proportion of Th9 cells in the peripheral blood of AITDs and GD groups compared to the NC group. IL-9 plasma level was correlated positively with TgAb in HT patients.

We did a correlation study between the production of Th9-related variables and thyroid function, thyroid autoantibodies, and main effector cytokines of other CD4+T cell subsets to further understand the potential function of Th9 cells in AITDs. We found that IL-9 plasma level was correlated positively with TgAb in HT patients. It is well known that Tg levels are also elevated in differentiated thyroid cancer patients (26). In our study, we ruled out thyroid cancer patients through clinical evaluation and diagnostic tests, such as through ultrasound, thyroid function tests, and fine needle aspiration biopsies when necessary. Therefore, we speculate that IL-9 may be related to the pathogenesis of HT.

The production of TRAb is a major contributor to the development and extrathyroidal manifestations associated with GD, and its accuracy is remarkable, with a 99.0% specificity and sensitivity (27). By monitoring the initial expression and TRAb titer changes, clinicians can effectively adjust treatment strategies, and TRAb is capable of accurately predicting the prognosis of GD (28). However, in our study, neither the expression of Th9-related factors nor the proportion of Th9 cells was associated with TRAb in GD patients. This is probably because refractory GD patients, who have been taking medication for a prolonged period, may experience a reduction in TRAb levels. It also should be noted that TRAb exerts different effector functions: besides the TSH stimulating action also the blocking activity and the one as growth factors (29). Until now, it is difficult to distinguish which TRAb is measured only by binding assays. Therefore, the relationship between IL-9 and TRAb still needs further study.

TPOAb is detected in 90-95% of individuals with HT, and can confirm the presence of autoimmunity and can also indicate the rate at which subclinical hypothyroidism will advance to overt hypothyroidism or a state that requires medical attention (30–32). Our investigation revealed a significant correlation between the proportion of Th9 cells in PBMCs of HT group and the titers of TPOAb. These findings imply a close association between Th9 cells and HT, potentially contributing to the production of TPOAb. In line with this, multiple studies have found a positive correlation between Th9 cells proportion or IL-9 expression and disease activity. For example, the increased serum IL-9 level is associated with the disease activity of UC and Crohn’s disease (11, 33–36). In addition, the significantly elevated proportion of Th9 cells was significantly increased in adolescents with type 1 diabetes mellitus and positively associated with HbA1c (37, 38). An increase in IL-9 levels and the proportion of Th9 cells was also found in SLE and related to disease activity and severity (10). Furthermore, in systemic sclerosis, heightened IL-9 expression in diseased skin could promote B cells to produce autoantibodies (39). These results indicate that Th9 cells may play a key role in the development of many autoimmune diseases.

Prior investigations have established that the upregulation of Th17 cells, primarily characterized by the secretion of IL-17 and IL-21, plays a significant role in the development of GD and HT (40, 41). Interestingly, we also found a positive association between mRNA expressions of IL-9 and IL-17 in GD group. And a positive relationship was also observed between IRF4 mRNA and IL-21mRNA in GD group. Meanwhile, in both AITDs and HT group, the expression of IRF4 mRNA was likewise positively associated with IL-17 mRNA. These findings provide additional confirmation of the involvement of Th9 cells in the pathogenesis of AITDs and suggest their potential to facilitate the differentiation of Th17 cells and the secretion of associated cytokines.

In alignment with these findings, a study investigating Behcet’s disease reported a favorable association between increased serum IL-9 levels and IL-17 (42). Furthermore, an augmented presence of Th9 cells and elevated plasma IL-9 levels in patients with active immune thrombocytopenia exhibited positive correlations with Th17 cells and IL-17 levels, respectively (43). These outcomes elucidate a robust interrelation between Th9 and Th17 cells. Remarkably, a separate study has delineated that a heightened prevalence of Th9 cells in the synovial tissue of rheumatoid arthritis (RA) patients is linked to disease activity, and that IL-9 can facilitate the Th17 differentiation (12). Nevertheless, within the context of autoimmune thyroid diseases (AITDs), the effect of IL-9 on Th17 cell differentiation remains obscure and necessitates further investigation.

To further investigate the role of IL-9, we cultured PBMCs from GD, HT, and NC groups *in vitro* and stimulated with human recombinant IL-9 protein. As we all know, an imbalance in the ratio of Th1/Th2 cells has a dramatic effect on the onset and extent of GD (44). Th1 cells primarily release IFN-γ, whereas Th2 mainly produce IL-4 (45). We found that IL-9 could promote the secretion of IFN-γ by PBMCs in both GD and NC groups. This suggests that IL-9 may promote Th1 cell immune response and secrete IFN-γ. Despite the absence of statistical difference, the IL-17 levels in the supernatant of PBMCs in HT group and NC group were higher than those before IL-9 protein intervention, further suggesting that IL-9 has the potential to promote the Th17 differentiation.

To further confirm the involvement of Th9 cells in the development of AITDs, we also performed SNP detection of IL-9 loci (rs31564, rs2069879, rs1859430 and rs2069868) in AITDs patients and controls, and analyzed the association between the polymorphisms of these loci and AITDs. Among them, the polymorphisms of rs1859430 and rs2069868 of IL-9 have been studied in GD patients of the Chinese Han population, but no correlation with GD was found (46). However, this study did not conduct model analysis and investigate the association between IL-9 gene polymorphisms and the incidence of HT. Consistent with the results of this study, no significant differences were found in the genotype and allele frequencies of IL-9 rs31564, rs2069879, rs1859430 and rs2069868 between AITDs, GD and HT and the control group. In order to more accurately evaluate the association of these four loci with AITDs, we made an association analysis under different genetic models for each locus. And it turned out that IL-9 rs2069879 polymorphism was significantly associated with AITDs, GD and HT under additive model. These findings suggest an association between IL-9 polymorphisms and the onset of AITDs. But it is important to recognize that additive models may have certain limitations in analyzing the complexity of genetic interactions. Therefore, future studies using more genetic models, functional analysis, more polymorphic sites of IL-9 and validation of different populations are essential to confirm the specific role of IL-9 polymorphism in the pathogenesis of AITDs.

## Conclusions

As an emerging CD4^+^T cell subset, Th9 may exert an important role in the development and progression of refractory GD and HT. IL-9 is expected to be a novel and potential therapeutic target for AITDs.

## Data availability statement

The original contributions presented in the study are included in the article/**Supplementary Material**. Further inquiries can be directed to the corresponding authors.

## Ethics statement

The studies involving humans were approved by the Ethics Committee of Zhoupu Hospital. The studies were conducted in accordance with the local legislation and institutional requirements. The participants provided their written informed consent to participate in this study.

## Author contributions

QY: Formal Analysis, Investigation, Writing – original draft. ZS: Formal Analysis, Visualization, Conceptualization, Writing – review & editing. BW: Investigation, Software, Writing – review & editing. PD: Investigation, Methodology, Writing – review & editing. QQ: Data curation, Software, Writing – review & editing. JZ: Supervision, Writing – review & editing. JZ: Funding acquisition, Project administration, Supervision, Writing – review & editing.

## Supplementary material

Supplementary Table 1 The number of samples in each part of the experiment.

Supplementary Table 2 Primer sequences for each gene.

Supplementary Table 3 Allele distributions of IL-9 loci in AITD patients and controls.

Supplementary Table 4 Allele distributions of IL-9 loci in GD patients and controls.

Supplementary Table 5 Allele distributions of IL-9 loci in HT patients and controls.
