# Supplementary material for: Increased interleukin-9 and Th9 cells in patients with refractory Graves’ disease and interleukin-9 polymorphisms are associated with autoimmune thyroid diseases
Source: Front Immunol. 2024 Mar 28;15:1341749. doi: 10.3389/fimmu.2024.1341749 (PMC11007129; doi:10.3389/fimmu.2024.1341749)
Supplement: Supplementary Table 1 — The number of samples in each part of the experiment. [file Table_1.docx]

**Supplementary Table 1 The number of samples in each part of the experiment**

| **Experiment** | **Number of each group** | | | |
| --- | --- | --- | --- | --- |
|  | AITD | GD | HT | NC |
| PCR assay | 62 | 38 | 24 | 24 |
| Cytokine detection | 47 | 24 | 23 | 21 |
| Flow cytometry | 51 | 28 | 23 | 16 |
| Cell culture | 11 | 6 | 5 | 6 |
| SNP | 985 | 623 | 362 | 724 |
| Total number | 1156 | 719 | 437 | 791 |

**Supplementary Table 2 Primer sequences for each gene**

| Gene | Forward | Reverse |
| --- | --- | --- |
| IL-9 | GACATCAACTTCCTC ATC AA | ATGCCCAAACAGAGACAA C |
| IRF4 | GCTGATCGACCAGATCGACAG | CGGTTGTAGTCCTGCTTGC |
| IFN-γ | TCCAACGCAAAGCAATACAT | GCAGGCAGGACAACCATTAC |
| IL-4 | GAAGAGAGGTGCTGATTG | GGAAGAACAGAGGGGGAAG |
| IL-17 | CAATCCCACGAAATCCAGGATG | GGTGGAGATTCCAAGGTGAGG |
| IL-10 | GACTTTAAGGGTTACCTGGGTTG | TCACATGCGCCTTGATGTCTG |
| IL-21 | CCAAGGTCAAGATCGCCACATG | TGGAGCTGGCAGAAATTCAGGG |
| IL-22 | TGCTGTTCCCTCAATCTG | TGTGCTTAGCCTGTTGCTG |
| β-actin | CATTGCCGACAGGATGCAG | CTCGTCATACTCCTGCTTGCTG |

**Supplementary Table 3 Allele distributions of IL-9 loci in AITD patients and controls**

| SNP | Allele | NC (%) | AITD (%) | P | OR | 95% CI |
| --- | --- | --- | --- | --- | --- | --- |
| rs31564 | T | 626 (43.2) | 871 (44.2) | 0.568 | 1.041 | 0.908-1.193 |
|  | G | 822 (56.8) | 1099 (55.8) |  |  |  |
| rs2069879 | G | 59 (4.1) | 67 (3.4) | 0.302 | 0.829 | 0.580-1.184 |
|  | C | 1389 (95.9) | 1903 (96.6) |  |  |  |
| rs1859430 | A | 59 (4.1) | 64 (3.2) | 0.200 | 0.791 | 0.551-1.134 |
|  | G | 1389 (95.9) | 1906 (96.8) |  |  |  |
| rs2069868 | A | 209 (14.4) | 284 (14.4) | 0.989 | 0.999 | 0.823-1.211 |
|  | G | 1239 (85.6) | 1686 (85.6) |  |  |  |

**Supplementary Table 4 Allele distributions of IL-9 loci in GD patients and controls**

| SNP | Allele | NC (%) | GD (%) | P | OR | 95% CI |
| --- | --- | --- | --- | --- | --- | --- |
| rs31564 | T | 626 (43.2) | 535 (42.9) | 0.878 | 0.988 | 0.848-1.151 |
|  | G | 822 (56.8) | 711 (57.1) |  |  |  |
| rs2069879 | G | 59 (4.1) | 41 (3.3) | 0.283 | 0.801 | 0.534-1.202 |
|  | C | 1389 (95.9) | 1205 (96.7) |  |  |  |
| rs1859430 | A | 59 (4.1) | 39(3.1) | 0.192 | 0.761 | 0.504-1.148 |
|  | G | 1389 (95.9) | 1207(96.9) |  |  |  |
| rs2069868 | A | 209(14.4) | 180 (14.4) | 0.993 | 1.001 | 0.807-1.242 |
|  | G | 1239(85.6) | 1066 (85.6) |  |  |  |

**Supplementary Table 5 Allele distributions of IL-9 loci in HT patients and controls**

| SNP | Allele | NC (%) | HT (%) | P | OR | 95% CI |
| --- | --- | --- | --- | --- | --- | --- |
| rs31564 | T | 626 (43.2) | 336 (46.4) | 0.160 | 1.137 | 0.95-1.36 |
|  | G | 822 (56.8) | 388 (53.6) |  |  |  |
| rs2069879 | G | 59 (4.1) | 26 (3.6) | 0.584 | 0.877 | 0.548-1.403 |
|  | C | 1389 (95.9) | 698 (96.4) |  |  |  |
| rs1859430 | A | 59 (4.1) | 25 (3.5) | 0.479 | 0.842 | 0.523-1.356 |
|  | G | 1389 (95.9) | 699 (96.5) |  |  |  |
| rs2069868 | A | 209(14.4) | 104 (14.4) | 0.966 | 0.994 | 0.771-1.282 |
|  | G | 1239(85.6) | 620 (85.6) |  |  |  |
